# Emotional and Cognitive Preservice Science Teachers’ Engagement While Living a Model-Based Inquiry Science Technology Engineering Mathematics Sequence About Acid-Base

**DOI:** 10.3389/fpsyg.2021.719648

**Published:** 2021-10-08

**Authors:** Luisa López-Banet, David Aguilera, M. Rut Jiménez-Liso, F. Javier Perales-Palacios

**Affiliations:** ^1^Department of Science Education, Faculty of Education, University of Murcia, Murcia, Spain; ^2^Department of Science Education, Faculty of Science Education, University of Granada, Granada, Spain; ^3^Sensociencia Team, CEIMAR-University of Almería, Almeria, Spain

**Keywords:** cognitive engagement, emotional engagement, gender roles, model-based inquiry (MBI), preservice chemistry secondary teachers, scientific methods, skills development, STEM-science technology engineering mathematics

## Abstract

Science inquiry and modeling activities have been proved to heighten emotional situations; therefore, research about emotions should aim to identify which activities promote student engagement with Science, Technology, Engineering and Mathematics fields through multidimensional models that include emotional and cognitive engagement. This research is focused on science teachers’ need to carefully review their classroom instructions to ensure that students are provided with opportunities to develop appropriate understandings of acid/base models (and their concepts). To achieve this, we have implemented a short model-based inquiry acid-base instructional sequence in the context of a TV-spot about chewing gum. A descriptive, non-experimental quantitative methodology with a heuristic (emotional: self-report questionnaire; and cognitive: self-regulation questionnaire) has been used to analyze what Pre-Service Secondary Education Teachers from several Spanish universities recognize to have learned and felt in each activity. Differences regarding knowledge declared by the participants were identified in all the tasks from before to after carrying them out. Furthermore, the results seem to indicate that there are significant relationships between the knowledge and the emotions, being different depending on the skill involved. Significant correlations between emotions have been found. However, there were no significant correlations with either rejection and knowledge or with other emotions, which points to emotional engagement. Generally, no significant differences were identified between emotions and gender or universities, with some exceptions between genders in two tasks. Thus, the results led us to reflect on the instructional sequence implementation’s ability to bring awareness to the learning process and how it produces multidimensional engagements.

## Introduction

Daily life situations in which people must use scientific knowledge of acids and bases are numerous and imply making decisions about certain actions that involve socio-scientific controversies in the face of unfounded advertisements about health or home remedies. Nevertheless, the scarcity of acid/base contents to explain everyday phenomena at levels below high school, both in the official curriculum and in textbooks (Jiménez-Liso et al., 2002), makes it difficult for teachers to see the need to introduce them to Secondary School or to have teaching resources to lean on. Therefore, a specific acid/base training for teachers that integrates these chemical contents with pedagogical content knowledge is essential. To achieve this, it is necessary to design instructional sequences of authentic practices for teachers, with a clear and recognizable teaching approach that connects this knowledge with everyday phenomena and that also promotes epistemological knowledge.

The role of emotions during teacher training has been scarcely researched even though the process of learning to be a science teacher is a situated social practice that is infused with emotion (Bellocchi et al., 2014). It has been proven that science inquiry and modeling activities can heighten emotions (Jimenez-Liso et al., 2019). We wanted, thus, to focus on measuring the instructional sequence designed for pre-service teachers on a combination of multiple dimensions of engagement. As Sinatra et al. (2015) mentioned, engagement in the scientific practices as a whole has not been extensively researched, and therefore the specific connections among the behavioral, cognitive, emotional, and agentic dimensions of engagement are speculative.

In this paper, we want to measure an integrated view of engagement that considers multiple dimensions in interaction (some of the dimensions of engagement overlap). Therefore, we address this issue by implementing and evaluating the Model-Based Inquiry (MBI in advance) instructional sequence’s effect on Pre-Service teachers’ (PSTs in advance) emotional and cognitive engagement related to acid/base contents as they develop professional competence in this field.

## Theoretical Framework

The teaching and learning of scientific practices in which students are engaged is emerging in many countries (Jaber and Hammer, 2016). Students learn not only the knowledge but also scientific skills in each practice, and they are able to recognize a broad spectrum of scientific methods rather than just one (Halawa et al., 2020). However, PSTs may not notice all of these benefits; because they lack prior professional experience, their spontaneous conceptions about teaching, which are rooted in their extensive experience as students, represent a real obstacle to change in teaching (Tobin and Espinet, 1989). To achieve substantial changes (Milner et al., 2012) the teaching approach itself must respond to the proposed MBI approach so that future teachers are trained following the same approach that they are to implement with their students (van Zee and Roberts, 2001; van Zee, 2006; Wee et al., 2007). In this sense, different studies have shown the importance and effectiveness of future teachers, during their training process, experiencing innovative instructional sequences that serve as a model teaching approach (Wandersee et al., 1994; Jimenez-Liso et al., 2021). Such instructional sequences consistent of integrated learning of content, teaching strategies, and students’ ideas. Some authors have gone further by suggesting that future teachers can participate in cycles of planning, teaching, and reflection on their experiences in the schools (Parker, 2006; Zembal−Saul, 2009).

Furthermore, chemistry is in a good position to contribute to achieving one of the United Nations (2021) Sustainable Development Goals (SDGs), that of gender equality, and to respond to UNESCO (2017) suggestion that science teachers can positively influence girls’ interest in STEM subjects. Chemistry can help shape young women’s future choice of professional STEM careers, from an inclusion perspective and can play a prominent role in the treatment of biases with a model of good systemic practices. The development of practical skills, training in cultural competence, and promotion of approaches aimed at improving equality, diversity and inclusion, should help to achieve the SDGs (Mehta et al., 2018). For these reasons, in this theoretical framework, we chose to focus on the MBI teaching approach, on justifying the need for an initial training of eminently practical teachers and on the cognitive and emotional dimensions that will be the object of measurement of the instructional sequence evaluation.

### Model-Based Inquiry

Many international research projects and reports opt for the MBI approach—which aims to promote the learning of scientific competence by involving students in the design and development of their own scientific research—due to its advantages of motivating students and favoring both the learning of science and the characteristics of scientific activity. The MBI model presents learning as a dynamic process aimed at building descriptive, explanatory, and predictive knowledge, and producing an evolution of the students’ ideas as they wonder about natural events (Khan, 2007; Windschitl et al., 2008; Schwarz, 2009). For this reason, inquiry and modeling are considered two of education’s purposes to which scientific disciplines must contribute to involve students in the practice of science.

Jiménez-Liso et al. (2018) described the implementation, among Secondary Education students, of a sequence of inquiry and modeling on the acid/base contents through the use of pH-meters that allow students to explain and predict outcomes based on acid/base phenomena. In addition to the conceptual learning objective, such as understanding the difference between dilution and neutralization, the sequence promotes the development of research skills, and helps students to be aware of the constructed procedural knowledge. Throughout the process, students are dedicated to raising questions and expressing, justifying, and discussing their ideas through different forms of communication (oral and written language, graphics, drawings, etc.), designing the search for tests to contrast their own ideas, analyzing results, and obtaining and discussing conclusions about the results and the involved processes. At the end, students recognize the need to use a model to explain the new phenomena and make a proposal that is jointly evaluated and reviewed.

### Preservice Teachers Training

Secondary chemistry teachers do not always include scientific practices in laboratory sessions (Boesdorfer and Livermore, 2018) or align classroom activity structures with the Next Generation Science Standards (NGSS) recommendations (Criswell and Rushton, 2014). Conceptions influenced by traditional practices, such as the lack of students’ participation in establishing their own scientific ideas, or a teacher’s excessive control over the completion of the task over the understanding of it, represent a limiting factor in teachers’ implementing activities by inquiry and modeling. For these reasons, improving teachers’ understanding could encourage more student inclusion in science classes (Donnelly et al., 2014). A guided inquiry and modeling instructional framework with science methods instruction can enable PSTs to apply their knowledge to enactments of reform-oriented science teaching approaches (Schwarz and Gwekwerere, 2007). It is recommended that teachers focus not only on content knowledge, but also on procedural and epistemic knowledge as well as on scientific understanding of phenomena with its proper interpretation (Bellová et al., 2017). During initial training, MBI approaches lead to PSTs becoming aware of how science is learned (both conceptual and procedural contents) and stating that they intend to implement scientific practices as future teachers (Jimenez-Liso et al., 2019). In order to offer an alternative teaching approach to what PSTs experienced as students, we selected an MBI instructional sequence that achieves that goal and that has been designed to require learning from STEM areas.

### Cognition and Emotions

Human behavior regarding approach or avoidance is determined not only by the reflective evaluation of its anticipated consequences, but also by affective impulses. Thus, emotion and behavior are related at least reflexively and impulsively (Strack et al., 2016). Therefore, we will pay special attention to studies based on emotional frameworks, which have begun to associate emotions with participation in challenging projects and creative problem solving.

It is essential to assume as part of teachers’ academic training that including the development of emotional skills has a great impact on their professional and personal enrichment (Valente and Lourenço, 2020). However, previous research studies have only emphasized the cognitive and behavioral components, excluding the emotional dimension, which currently constitutes a line of research in science education that is in constant growth and is in urgent need of examination (Fortus, 2014; Mellado et al., 2014; Bellocchi et al., 2015; Zembylas, 2016). Emotions are present throughout engagement with science practices, both students and scientists, which thus calls for pursuing deeper understanding (Jaber and Hammer, 2016). It is essential to train emotionally competent teachers who know how to diagnose and self-regulate their emotions (Bisquerra Alzina and Pérez Escoda, 2007). The first teaching experiences are emotionally very strong and can set behavioral strategies (Mellado et al., 2014). Disciplinary engagement in science is characterized by the experience of epistemic affect, such as the excitement of having a new idea or the irritation at an inconsistency (Jaber and Hammer, 2016). Jimenez-Liso et al. (2019) made PSTs aware of positive emotions, like satisfaction or interest experienced when they were “doing scientific practices.” Therefore, limiting teaching and learning to methods solely aligned with a cognitive, rational perspective, without emotive elements of learning, could be unproductive whereas aesthetic experiences promote more science learning (Girod et al., 2010).

Gottlieb et al. (2018) determined that awe and scientific thinking are positively associated; thus, engaging in science requires a disposition to revise beliefs about seeing new evidence. Awe is considered the emotional state most likely to impact outcomes in science learning (Valdesolo et al., 2017) and implies the accommodation of new information that cannot be assimilated into preexisting schemas (Keltner and Haidt, 2003), according to Piagetian theories of cognition (Piaget, 1971). Moreover, when a sudden awareness of connections between concepts arises without a conscious understanding of the processes then the tacit processing to reach a conscious outcome is allowed. For tacit processing to achieve a conscious result, students need to be given enough time to engage in problem solving, or to think about answers to questions in class, which time is essential to their becoming aware of how they learn science (Brock, 2017).

## Objective

To analyze the PSTs perceptions of students’ learnings after they experience this sequence of activities by inquiry and modeling, we need the PSTs to become aware of the importance of linking the cognitive and the affective in learning. Thus, we propose having groups of teachers implement a sequence of inquiry and modeling on the effects of chewing gum on the mouth’s pH level to focus the PSTs on the teaching approach at the same time while they, themselves, learn and reflect on what they learned: how they learned and how they felt during the instructional sequence implementation.

In addition, this implementation will allow us to comprehensively gauge their participation in both the cognitive and emotional dimensions.

This research is focused on science teachers’ need to carefully review their classroom instruction to ensure that PSTs are provided with opportunities to develop appropriate understandings of the acid/base models (and their concepts). Thus, the main objective of this research is to allow future teachers to become aware of the facts of acid/base contents (self-regulation of learning), how they learn (leading to an explicit discussion on the phases of inquiry and modeling), how they help others to learn, and their relationship with emotions (what emotions emerge during the implementation practice).

## Method

### Description of the Instructional Sequence

To achieve the abovementioned aims, two teachers (first and third authors of this paper) implemented a short MBI acid-base instructional sequence (length 2 h) in the context of a TV-spot about chewing gum (López-Banet et al., 2021). The implementation was composed of ten tasks or key moments that PSTs responded to individually before sharing and discussing them with the whole class. T1–T9 are included in Table 1, and T10 consists of the self-regulation of learning and feelings. The sequence described above requires coordinating diverse disciplines that constitute the STEM field (Martín-Páez et al., 2019), by encompassing chemical contents (Science), the understanding of a logarithmic scale (Mathematics) through a dynamic visualization with sensors (Technology) and generating a variety of experimental designs (Engineering). Furthermore, both the interpretation of several types of resources (such as textual, visual, or sound) and the skill of creating an explanatory model for a scientific question, leads to a natural connection with the visual arts. Thus, a STEAM view is needed from the interdisciplinary of the teaching proposal, which is further enriched by the incorporation of knowledge and skills from all areas (López-Banet et al., 2021).

**TABLE 1:** Knowledge declared by the participants for the 9 tasks, before and after carrying them out.

Task (before/after)	N	Minimum	Maximum	Median	Mean	Standard deviation	Evolution*	Z	.p	ES
T1: What are acid substances (before)	45	2	5	3	2.80	0.869	1.2	−5.259	<0.01	1.353
T1 (after)	45	2	5	4	4.00	0.905				
T2: How can we prove that they are acidic? (before)	45	1	5	3	3.09	0.973	1.27	−5.203	<0.01	1.445
T2 (after)	45	2	5	5	4.36	0.773				
T3: Hypothesis on the effect of chewing gum on the pH of the mouth (before)	45	1	5	1	1.58	0.783	2.49	−5.478	<0.01	2.616
T3 (after)	45	1	5	4	4.07	1.095				
T4: Design and evaluate experiments to test your hypothesis (before)	45	1	5	2	2.20	0.944	1.53	−5.597	<0.01	1.530
T4 (after)	45	2	5	4	3.73	1.053				
T5: Chewing gum pH data analysis: coincidences and discrepancies (before)	45	1	5	2	1.80	0.919	2.13	−5.597	<0.01	2.207
T5 (after)	45	1	5	4	3.93	1.009				
T6: If chewing gum dilutes acids or neutralizes them (before)	45	1	5	1	1.56	0.841	2.53	−5.704	<0.01	2.641
T6 (after)	45	1	5	4	4.09	1.062				
T7: The mathematical zoom: liters of saliva to “neutralize” the acids in the mouth (before)	45	1	5	1	1.62	0.886	2.34	−5.709	<0.01	2.510
T7 (after)	45	1	5	4	3.96	0.976				
T8: “Pac-man” model to explain why the pH drops in the mouth after chewing Orbit gum (before)	45	1	5	1	1.47	0.842	2.95	−5.862	<0.01	3.759
T8 (after)	45	2	5	5	4.42	0.723				
T9: “Pac-man” model to explain why the balloon is inflated (baking soda and vinegar). Make sense of the chemical formulation (before)	45	1	5	1	1.58	0.839	2.71	−5.770	<0.01	3.222
T9 (after)	45	2	5	4	4.29	0.843				

*Evolution in the average of the knowledge perceived in each item of the questionnaire KPSI (before and after).

### Research Sample

Two groups of PSTs in training participated in this study. They were students of a two-semester academic calendar master’s degree at two public universities, which is compulsory for earning a certification for secondary school teaching in Spain. Both groups consist in a variety of students participating in the same MBI sequence that it was taught by two teachers with different profiles of their respective master’s degrees, at University of Almería and Murcia (different training plan), in the same academic term (2020/21). These circumstances are unique and prevent from having a greater sample size. It is worth mentioning that the participants had never experienced MBI instructional sequences when they enrolled in this master’s program.

The first group was 27 PSTs (18 women and 9 men) from the University of Almería, who had previously studied Biology (11), Biotechnology (5), Chemistry (2), Biochemistry (2), Environmental sciences (2), Food Technology (2), Geology (2) and Pharmacy (1). The second group had 18 students (11 women and 7 men) from the University of Murcia, who had pursued Chemistry (6), Physics (6), Biochemistry (4), Chemical Engineering (1) and Food Technology (1).

### Instrument for Evaluating Pre-Service Teachers Engagement

In order for PSTs to notice the what and how of learning as well as what emotions they felt, at the end of the instructional sequence we added *in situ* measures of the self-regulations of learnings and emotions with a questionnaire that has a minimal disruption of the flow of PSTs learnings, because it is a task (T10) inside of the instructional sequence with coherence and sense to teachers and students (Jimenez-Liso et al., 2021). Emotional engagement is addressed by the presence of emotions associated with the learning outcomes (such as interest and concentration) and the absence of emotions that would hinder the task (rejection). The cognitive dimension refers to all the strategies that students must develop in order to build significant learning processes, including self-regulation of learning.

We used a descriptive, non-experimental quantitative methodology using a Knowledge and Prior Study Inventory (KPSI) as self-regulation questionnaire (Tamir and Amir, 1981; Jimenez-Liso et al., 2019) and emotions recognized by PSTs in the key moments of the applied inquiry sequence. The KPSI consists of a self-report on ten emotions: nine of them (rejection, concentration, insecurity, interest, boredom, confidence, satisfaction, dissatisfaction, and bashfulness) were in a previously validated questionnaire (Jimenez-Liso et al., 2019). We also included another emotion (surprise) because surprising observations could later elicit the “aha” or awe moment, which scientists have linked to the learning process and to situations of discovery (Cuzzolino, 2021). PSTs were able to select the emotion label related to all the key moments following the sequential order of the model-inquiry sequence. In order to self-regulate the knowledge, before and after the sequence was implemented, PSTs answered on a scale of 1 to 5 (1: I know nothing; 2: I know a little; 3: I know well; 4: I know it very well; and 5: I can explain it to a friend) for each key moment, in chronological order.

### Data Analysis

A descriptive analysis was carried out for the knowledge variable (minimum, maximum, median, mean, and standard deviation) and a frequency analysis for the different emotions under consideration. Non-parametric tests were also applied, as the sample size as well as the nature of the data recommend it. The U-Mann Whitney test was used to determine significant differences among independent samples, according to the university to which the students belonged and to their gender. The Wilcoxon W test was used to verify the significance of the differences between the pre and post results of the knowledge variable. The Spearman correlation coefficient was calculated to determine the relationship between the knowledge reached by the students in the post-test phase and the emotions experienced during the experience. Also, in order to determine the relationship between gender and emotions, Pearson’s Chi-square was calculated. Finally, Cohen’s d (effect size, ES) was calculated for the knowledge variable. The indications of Cohen (1988) were followed to interpret the ES.

## Results

No significant differences in knowledge were identified based on gender nor based on the students’ universities in any of the tasks, with the exception of T2 (How can we prove that they are acidic?). Specifically, at the time prior to performing the second task, there were significant differences (*p*. = 0.005) between the University of Murcia (mean = 3.61; Standard dev. = 0.979) and the University of Almería (mean = 2.74; Standard dev. = 0.813), being equated after the development of the activity, at which time there were no significant differences. This data could be due to previous academic training, since at the university in which a lower initial value was obtained, they have more varied degrees, the majority being from the field of biology, while practically all the university students who obtained a higher value had degrees whose contents were closer to those dealt with in the sequence. These factors could explain the higher initial value. For example, those with the qualifications of biology, environmental sciences, or geology gave scores of less than 3 on knowing this task, while those with degrees in chemistry, physics, and biotechnology gave scores of greater than 3 (Chemistry graduates averaged scores were 3.88). Table 1 includes the values on their learning, which were perceived by the participants regarding each task.

**FIGURE 1:**
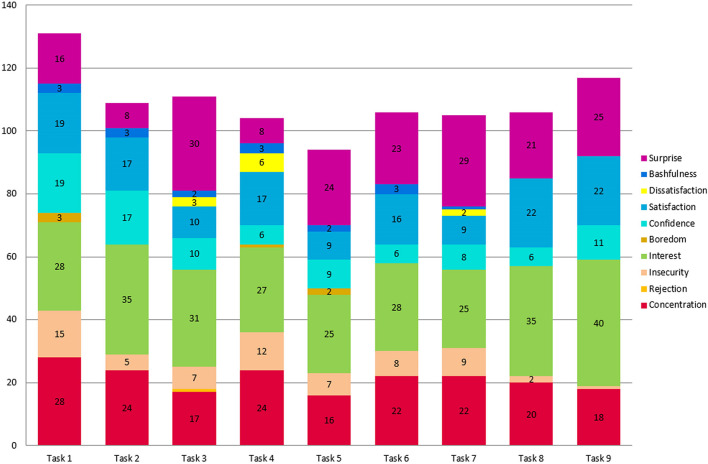
Emotions experienced by the 45 participants of UAL and UMU.

On the other hand, significant differences were identified in the nine tasks from before to after carrying them out (Table 1), with an increase of more than 2.5 after completing the following tasks: 6 (If chewing gum dilutes acids or neutralizes them); 8 (the “Pac-man” model to explain why the pH drops in the mouth after chewing Orbit gum); and 9 (“Pac-man” model to explain why the balloon is inflated), which increase seems to indicate that the PSTs perceived that they learned new knowledge about the effect of chewing gum, as well as they developed a model to explain what happens (Figure 1). In addition, the obtained ESs indicate substantial progress in terms of the students’ perceived knowledge gains (Table 1).

Regarding the emotions they manifested throughout the sequence, in general there was a high interest in the activity as well as concentration and surprise (Figure 1).

Generally, no significant differences were identified between emotions and gender, with the following exceptions: Women (*N* = 19) manifested concentration to a greater extent than did men (*N* = 9) in task 1 (χ^2^ = 4.543; *p*. = 0.033); and in task 4, women (*N* = 10) also manifested greater insecurity (χ^2^ = 5.114; *p*. = 0.024) and greater dissatisfaction (women *N* = 6; men *N* = 0) (χ^2^ = 5.538; *p*. = 0.019).

With regards to the differences between universities, the students from UAL (*N* = 12) showed significantly more insecurity than did those from UMU (*N* = 0) (χ^2^ = 10.909; *p*. = 0.001) and more dissatisfaction (UMU *N* = 0; UAL *N* = 6) (χ^2^ = 4.615; *p*. = 0.032) in task 4 (Design and evaluate experiments to test your hypothesis). In task 5 (Chewing gum pH data analysis), UAL students (*N* = 7) showed significantly more insecurity than did those from UMU (*N* = 0) (χ^2^ = 5.526; *p*. = 0.019). In task 6 (If chewing gum dilutes acids or neutralizes them) the UAL students (*N* = 8) showed more insecurity than did those from UMU (*N* = 0) (χ^2^ = 6.486; *p*. = 0.011), while those from UMU (*N* = 13) exhibited more surprise than did those from UAL (*N* = 10) (χ^2^ = 5.351; *p*. = 0.021). Likewise, in task 6 the UAL students (*N* = 6) showed more confidence than did UMU students (*N* = 0) (χ^2^ = 4.615; *p*. = 0.032). Finally, in task 8 (“Pac-man” model to explain why the pH drops in the mouth after chewing gum) the UAL students (*N* = 6) experienced a greater degree of confidence than did the UMU students (*N* = 0) (χ^2^ = 4.615; *p*. = 0.032). It is worth mentioning that these results should be taken with caution since some of the significant differences may be due to the small number of students participating in the study. Thus, some of the emotions identified by UAL students have not been identified by any of the UMU (confidence, in tasks 6 and 8, insecurity in tasks 4, 5, and 6, dissatisfaction in task 4), and significant differences may result as a consequence of the fact that the UMU group is smaller.

Regarding the correlations between emotions and knowledge (Table 2), emotions were coded dichotomously (appearing or not appearing).

**TABLE 2:** Correlations between knowledge declared by the 45 participants (posttest) and emotions.

Task		Concentration	Rejection	Insecurity	Interest	Boredom	Confidence	Satisfaction	Dissatisfaction	Bashfulness	Surprise
T1	Knowledge	0.011	.	−0.317*	–0.084	−0.327*	0.352*	–0.061	.	–0.153	0.006
T2	Knowledge	0.023	.	−0.400**	–0.098	.	0.384**	0.144	.	−0.454**	–0.017
T3	Knowledge	0.258	0.149	–0.045	0.030	.	0.202	0.088	−0.407**	−0.319*	0.062
T4	Knowledge	0.073	.	–0.231	0.065	0.030	0.502**	0.240	−0.455**	–0.093	–0.218
T5	Knowledge	0.193	.	–0.203	0.065	–0.183	0.133	0.133	.	−0.297*	–0.110
T6	Knowledge	-0.183	.	–0.189	0.021	.	0.156	0.254	.	0.029	0.051
T7	Knowledge	0.029	.	−0.543**	–0.002	.	0.271	–0.036	0.254	–0.171	0.186
T8	Knowledge	0.093	.	–0.107	0.083	.	0.124	0.298*	.	.	0.054
T9	Knowledge	0.157	.	–0.95	0.298*	.	–0.037	0.189	.	.	0.171

*The correlation is significant at the 0.05 level (bilateral). **The correlation is significant at the 0.01 level (bilateral).

The results indicate that the relationship between knowledge and emotions declared by PSTs were different depending on the skill involved, as shown in Table 2. Nevertheless, it seems that concentration, rejection, and surprise are not significantly correlated to knowledge. Thus, in order to go further, significant correlations between emotions have been found in 8 of the 9 tasks (Table 3).

**TABLE 3:** Correlations between emotions declared by the 45 participants (posttest).

	Concentration	Rejection	Insecurity	Interest	Boredom	Confidence	Satisfaction	Dissatisfaction	Bashfulness	Surprise
Concentration										
Rejection										
Insecurity	T4: −0.363*									
Interest	T5: −0.478** T7: −0.427** T8: −0.371*		T5: −0.384**							
Boredom			T1: −0.378*	T1:0.343*						
Confidence			T1:0.318*							
Satisfaction	T7: −0.400**			T7: −0.335*		T6: −0.528** T7: −0.349*				
Dissatisfaction			T3: −0.377* T4: −0.355*				T4:0.306*			
Bashfulness			T2: −0.472** T3: −0.502** T4: −0.443** T6: −0.342* T7: −0.302*			T7: −0.324*	T1: −0.313* T7: −0.302*	T3: −0.807**		
Surprise				T1: −0.387**		T5:0.423**				

*The correlation is significant at the 0.05 level (bilateral); **The correlation is significant at the 0.01 level (bilateral).

Regarding the abovementioned emotions that are not correlated to knowledge, concentration appears to be negatively correlated to insecurity when the PSTs designed and evaluated experiments. Also, these emotions were negatively correlated with interest using several skills, such as analyzing data, figuring out the liters of saliva to “neutralize” the acids in the mouth and using a model to explain why the pH drops in the mouth after chewing the gum. Finally, concentration was also negatively correlated to satisfaction when they were calculating the liters of saliva. Thus, depending on the skill, concentration could be related to less insecurity, or interest or satisfaction. On the other hand, surprise was negatively correlated to interest when PSTs had to define an acid, thus they who acknowledged less knowledge manifested a greater surprise after the implementation of the sequence. Additionally, surprise and confidence were positively correlated with the analyzed data, which result could mean that they who were surer about this skill were more surprised when they realized the results. Finally, there were no significant correlations with either rejection and knowledge or with other emotions, which points to emotional engagement.

## Conclusion

According to studies based on emotional frameworks, student engagement with school science and modeling activities is multidimensional (emotional, cognitive, and behavioral) (Mellado et al., 2014; Bellocchi et al., 2015; Jimenez-Liso et al., 2021). In the study reported in this article, special attention has been paid to analyzing the learning perceived by PSTs after experiencing an acid-base sequence of activities by inquiry and modeling to promote their awareness of the importance of the affective in learning.

In this paper, the results seem to indicate that there are significant relationships between the knowledge and the emotions expressed by the participants in all of the activities. In some tasks, expressing greater confidence or satisfaction was positively related to greater perceptions of learning. Moreover, the students who considered they had learned more declared that they felt emotions that imply security in relation to skills as responding to the problem, proposing an experimental design to solve it, as well as explaining what happens through the use of a model. In the case of using a model, knowledge would be related to greater satisfaction and interest, which result is similar to that of previous studies where PSTs declared satisfaction or interest when they were “doing scientific practices” (Jimenez-Liso et al., 2019). However, those who showed less learning mentioned negative emotions in tasks that involved skills, such as making hypotheses, developing experimental designs, and conducting data analysis. Moreover, in each of these tasks where PSTs recognized different emotions, some of them showed significant correlations between them.

These results constitute a sample of the nature of PSTs’ emotional engagement with scientific practices considering diverse previous academic training and the students’ gender. On the one hand, although previous formation would affect their initial ideas, the results are similar for the rest of the tasks. Moreover, the implementation of the STEM sequence with PSTs did not show significant differences by gender, neither in the perceived knowledge nor in their expressed emotions. Thus, the involvement of both boys and girls in sequences with meaning and emotional commitment constitutes a possibility to alleviate the gender gap in STEM studies.

Furthermore, previous studies have found that participants who recognized the usefulness of scientific practices for supporting science learning stated their intention to implement them as future teachers (Jimenez-Liso et al., 2019). Thus, we can conclude that the acid/base MBI instructional sequence that was put into practice with both groups of PSTs might help them to notice the connection between theory-practice and to become conscious of the importance of including the scientific practices as part of the teaching and learning process.

Finally, as the main limitation of this research is the sample size, future perspectives would include teaching the same MBI sequence to more participants enrolled in similar master’s programs. This enlargement would allow not only to compare the obtained results but also to extend the conclusions regarding emotional engagement indicated by the present results.

## Data Availability Statement

The raw data supporting the conclusions of this article will be made available by the authors, without undue reservation.

## Ethics Statement

The participants provided their written informed consent to participate in this study according to the Research Ethics Commission of the University of Murcia.

## Author Contributions

LL-B drafted the manuscript and implemented the proposal at University of Murcia (Spain). DA performed all the statistical determinations and analyzed data. MRJ-L designed the research and implemented the proposal at University of Almería (Spain). FJP-P discussed the results and revised the manuscript. All authors ensure that evidence-based claims were made, revisions to logical sequencing of ideas, editing the manuscript drafts, and conceptual input.

## Conflict of Interest

The authors declare that the research was conducted in the absence of any commercial or financial relationships that could be construed as a potential conflict of interest.

## Publisher’s Note

All claims expressed in this article are solely those of the authors and do not necessarily represent those of their affiliated organizations, or those of the publisher, the editors and the reviewers. Any product that may be evaluated in this article, or claim that may be made by its manufacturer, is not guaranteed or endorsed by the publisher.
